# Digital PCR analysis of plasma cell-free DNA for non-invasive detection of drug resistance mechanisms in EGFR mutant NSCLC: Correlation with paired tumor samples

**DOI:** 10.18632/oncotarget.5068

**Published:** 2015-08-17

**Authors:** Hidenobu Ishii, Koichi Azuma, Kazuko Sakai, Akihiko Kawahara, Kazuhiko Yamada, Takaaki Tokito, Isamu Okamoto, Kazuto Nishio, Tomoaki Hoshino

**Affiliations:** ^1^ Division of Respirology, Neurology, and Rheumatology, Department of Internal Medicine, Kurume University School of Medicine, Kurume, Fukuoka, Japan; ^2^ Department of Genome Biology, Kinki University Faculty of Medicine, Osaka, Japan; ^3^ Department of Diagnostic Pathology, Kurume University Hospital, Kurume, Fukuoka, Japan; ^4^ Center for Clinical and Translational Research, Kyusyu University Hospital, Fukuoka, Japan

**Keywords:** T790M mutation, cell-free DNA, digital PCR, EGFR, non-small-cell lung cancer

## Abstract

As the development of resistance to epidermal growth factor receptor (EGFR) tyrosine kinase inhibitors (TKIs) has become an issue of concern, identification of the mechanisms responsible has become an urgent priority. However, for research purposes, it is not easy to obtain tumor samples from patients with *EGFR* mutation-positive non-small-cell lung cancer (NSCLC) that has relapsed after treatment with EGFR-TKIs. Here, using digital PCR assay as an alternative and noninvasive method, we examined plasma and tumor samples from patients with relapsed NSCLC to establish the inter-relationships existing among T790M mutation, activating *EGFR* mutations, *HER2* amplification, and *MET* amplification. Paired samples of tumor and blood were obtained from a total of 18 patients with NSCLC after they had developed resistance to EGFR-TKI treatment, and the mechanisms of resistance were analyzed by digital PCR. Digital PCR analysis of T790M mutation in plasma had a sensitivity of 81.8% and specificity of 85.7%, the overall concordance between plasma and tissue samples being 83.3%. *MET* gene copy number gain in tumor DNA was observed by digital PCR in three patients, of whom one exhibited positivity for *MET* amplification by FISH, whereas no patient demonstrated *MET* and *HER2* copy number gain in plasma DNA. Digital PCR analysis of plasma is feasible and accurate for detection of T790M mutation in NSCLC that becomes resistant to treatment with EGFR-TKIs.

## INTRODUCTION

Non-small-cell lung cancer (NSCLC) is the leading cause of cancer death worldwide [1]. Recent therapeutic strategies for NSCLC have focused on the development of molecular targeted therapies. Somatic mutations in the epidermal growth factor receptor (*EGFR*) gene have been identified as a major determinant of the clinical response to treatment with EGFR tyrosine kinase inhibitors (EGFR-TKIs) such as gefitinib, erlotinib and afatinib in individuals with NSCLC [2–7]. Although patients with *EGFR* mutation-positive NSCLC typically show good responses to EGFR-TKIs, resistance eventually develops after 9 to 14 months. Several mechanisms of acquired resistance to EGFR TKIs have been identified, including a second-site point mutation that substitutes methionine for threonine at position 790 (T790M) in the EGFR, amplification of the mesenchymal-epithelial transition (*MET*) proto-oncogene and human epidermal growth factor receptor 2 (*HER2*), and small-cell lung cancer transformation [8–10]. The most common event responsible for resistance is acquisition of the T790M mutation, which occurs in over 50% of patients who initially respond to EGFR-TKIs [8]. Current clinical approaches for overcoming resistance to EGFR-TKIs in NSCLC include the use of mutant-selective inhibitors of EGFR, a combination of cetuximab and afatinib, and a combination of EGFR-TKI with a drug inhibiting a resistance pathway, for example erlotinib used together with a MET inhibitor [10–14].

Although tumor samples are required for studying the mechanism of resistance to EGFR-TKIs, it is difficult to obtain such samples from patients with NSCLC who have acquired such resistance. The level of tumor cell-free DNA (cfDNA) in plasma specimens is reportedly higher in patients with lung cancer than in those without cancer [15]. Here, we evaluated the application of digital PCR analysis to plasma samples as an alternative and non-invasive method for investigating the mechanisms of resistance to EGFR-TKIs, including T790M resistance mutation and amplification of *MET* and *HER2*, in comparison with results obtained using tumor samples.

## RESULTS

### Patient characteristics and efficacy of EGFR-TKI treatment

Patient characteristics are shown in Table 1. Among the 18 patients, 16 were female and 2 were male. Seven patients had E746-A750 deletion in exon 19, 10 had L858R point mutation in exon 21, and one had a minor mutation involving S752-I759 deletion in exon 19. One patient received elrotinib and the others received gefitinib as initial EGFR-TKI treatment. The EGFR-TKI treatment was provided as the first-line chemotherapy in 13 patients, the second line in three, the third line in one, and the fourth line in one. The overall rate of response to initial EGFR-TKI treatment was 72.2%, with a median progression-free survival 11.7 months. Tumor specimens after acquisition of resistance to EGFR-TKI treatment were derived from the primary lung lesion in 7 patients, pleural effusion in 8, lymph node metastasis in 2, and pericardial effusion in one.

**Table 1:** Patient and tumor characteristics

Number	18
Age (years)	
Median	66
Range	50–81
Sex	
Male	2
Female	16
Smoking status	
Current/Former	1
Never	17
Histology	
Adenocarcinoma	18
Type of *EGFR* mutation	
E746-A750 del	7
L858R	10
S752-I759 del	1
Initial TKI treatment	
Gefitinib	17
Erlotinib	1
TKI treatment line	
1st	13
2nd	3
3rd	1
4th	1

Abbreviations: EGFR, epidermal growth factor receptor; TKI, tyrosine kinase inhibitor.

### Consistency of primary activating EGFR mutation between tumor and plasma

The results relating to the consistency of primary active EGFR mutation status with tumor and plasma cfDNA are shown in Table 2 and summarized in Table 3. One patient (Case 1) who had a minor mutation involving S752-I759 deletion in exon 19 was not evaluable. Of the remaining 17 patients, 15 had detectable activating EGFR mutation in the tumor, whereas 10 patients exhibited this in the plasma cfDNA. All mutation types in these patients were consistent with the primary EGFR mutation status detected before treatment.

**Table 2:** Analysis of resistance mechanism to EGFR-TKI treatment in tumor samples and plasma samples

Case	Primary EGFR mutation	site	TKI	Primary mutation		T790M mutation		MET		
				Tumor	Plasma	Tumor	Plasma	Tumor	FISH	Plasma
1	S752-I759 del	PE	E	NE	NE	p	p	n	n	n
2	E746-A750 del	PE	G	p	p	p	p	n	n	n
3	E746-A750 del	Lung	G	n	n	n	n	n	n	n
4	L858R	Lung	G	p	n	n	n	p	n	n
5	E746-A750 del	Lung	G	p	p	p	p	n	n	n
6	L858R	PE	G	n	n	n	p	n	n	n
7	L858R	PE	G	p	p	n	n	n	n	n
8	L858R	PCE	G	p	n	p	p	n	n	n
9	L858R	LN	G	p	p	n	n	p	p	n
10	L858R	Lung	G	p	p	p	n	n	n	n
11	L858R	Lung	G	p	p	p	p	n	n	n
12	L858R	PE	G	p	p	p	p	n	n	n
13	L858R	PE	G	p	n	p	n	n	n	n
14	E746-A750 del	Lung	G	p	n	n	n	n	n	n
15	L858R	LN	G	p	n	n	n	n	n	n
16	E746-A750 del	Lung	G	p	p	p	p	p	n	n
17	E746-A750 del	PE	G	p	p	p	p	n	n	n
18	E746-A750 del	PE	G	p	p	p	p	n	n	n

Abbreviations: EGFR, epidermal growth factor receptor; site, re-biopsy site; TKI, tyrosine kinase inhibitor; FISH, fluorescence in situ hybridization; PE, pleural effusion; PCE, pericardial effusion; LN, lymph node; E, erlotinib; G, gefitinib; NE, not evaluated; p, positive; n, negative.

**Table 3:** Concordance of primary *EGFR* mutation and T790M mutation between tumor samples and plasma samples

	Tumor					
	Primary mutation	Positive	Negative	Sensitivity	Specificity	Concordance
Plasma	Positive	10	0	66.7%	100%	70.6%
	Negative	5	2			
	Total	15	2			
	**T790M mutation**	**Positive**	**Negative**	**Sensitivity**	**Specificity**	**Concordance**
Plasma	Positive	9	1	81.8%	85.7%	83.3%
	Negative	2	6			
	Total	11	7			

One patient who had a minor mutation involving S752-I759 deletion in exon 19 was not evaluated for primary *EGFR* mutation.

### Consistency of T790M mutation between tumor and plasma

Ten T790M mutations were detected from all 18 plasma specimens, whereas 11 T790M mutations were found in the paired tumor samples (Table 2). Notably, one patient with plasma cfDNA T790M mutation had no T790M mutation in the corresponding tumor DNA sample, and two patients with T790M mutations in tumor DNA specimens had no T790M mutation in the corresponding plasma. The sensitivity and specificity of digital PCR analysis for T790M mutation in plasma was 81.8% and 85.7%, respectively, and the overall concordance between plasma and tumor samples was 83.3% (15/18). The correlation between T790M mutations detected in the plasma and tumor samples is summarized in Table 3.

### Detection of *MET* and *HER2* copy number

Analysis of tumor samples confirmed a gain of the *MET* copy number in three patients. However, FISH analysis demonstrated *MET* amplification in only one patient (Case 9), and no patients showed an increase of the *MET* copy number in plasma. *HER2* copy number gain in the tumor and cfDNA were analyzed by digital PCR. However, no copy number gain was detected in any of the 18 cases (data not shown).

## DISCUSSION

In this study using digital PCR assay as an alternative and non-invasive method for examining plasma and tumor samples, we investigated correlations among T790M mutation, activating *EGFR* mutations, *HER2* amplification, and *MET* amplification in patients with NSCLC relapse after treatment with EGFR-TKIs. Although previous studies have examined various techniques for non-invasive detection of *EGFR* mutations in NSCLC patients, such as amplification refractory mutation systems, denaturing high-performance liquid chromatography, multi-threaded electronic polymerase chain reaction, and direct sequencing, the results have been inconclusive, with sensitivities ranging from 43.1% to 81.2% [16–23]. Yung et al. demonstrated that digital PCR analysis had a high sensitivity of 91.7% for detection of *EGFR* mutation in plasma samples [24], suggesting that it would be a promising method for T790M analysis of plasma cfDNA. Digital PCR is both a qualitative and quantitative method, being capable of detecting genetic alterations with a high specificity of up to 0.001% [25]. It has been reported that digital PCR analysis has high sensitivity and a high detection ratio in comparison with an allele-specific PCR technique such as scorpion-ARMS [26]. Given the high rates of false negativity for *EGFR* mutation in plasma cfDNA [27], we investigated the utility of digital PCR analysis as a highly sensitive assay for clarifying the mechanism of resistance to EGFR-TKIs. Although other researchers have investigated the detection of T790M mutant alleles in plasma cfDNA, they mostly focused on factors predictive of EGFR-TKI treatment outcome, such as monitoring of T790M mutation [28–31]. In this study, we found that digital PCR analysis of T790M mutation in plasma had a high sensitivity of 81.8% and a specificity of 85.7%, and that the overall concordance between plasma and tissue samples was 83.3%. Using a highly accurate method of digital PCR analysis, our study demonstrated relatively high concordance with previous studies that had investigated EGFR mutation status in pre-treatment samples [16–23]. These results suggest that digital PCR may be a feasible and clinically applicable method for detection of T790M mutation in plasma cfDNA. As far as we are aware, no previous study has demonstrated a significant correlation of T790M mutation between tumor samples and plasma cfDNA in individuals with EGFR-mutant NSCLC that has acquired resistance to EGFR-TKIs. Recently, third-generation EGFR-TKIs have yielded promising results in patients with EGFR-mutation positive NSCLC resistant to existing EGFR-TKIs [11, 12]. These new drugs were designed to inhibit both the activating EGFR mutation in patients with NSCLC as well as the T790M resistance mutation, and showed a high positive response rate to T790M mutation-positive NSCLC of approximately 60% in phase I clinical trials [32]. Although it is necessary to accurately identify T790M mutation-positive patients for proper use of these drugs, there are untestable patients such as those in whom tumor lesions cannot be acquired safely, or those who do not consent to re-biopsy. Given the high sensitivity and specificity of T790M detection in plasma samples, digital PCR analysis for T790M may be a promising approach for *EGFR* mutant NSCLC that has relapsed after EGFR-TKI treatment.

We also analyzed the plasma cfDNA of primary activating *EGFR* mutation, which had been detected previously in tumors before EGFR-TKI treatment, in matched samples obtained after acquisition of resistance to EGFR-TKI. Our results revealed that primary activating *EGFR* mutation in plasma had a sensitivity of 66.7%, which was relatively low in comparison to those in previous studies that had examined EGFR mutations in pre-treatment plasma [24], and those for the T790M mutation in the present study. One possible explanation for these discrepancies may be tumor mutational heterogeneity. Several previous studies of NSCLC have demonstrated a discrepancy of *EGFR* mutation status between the primary tumor and the corresponding metastatic lesions [33–35], and it has been suggested that the *EGFR* mutation status in the latter may be more associated with favorable efficacy of EGFR-TKI treatment than that in the former [35]. Discordance of *EGFR* mutation status has also been reported even among different parts of a primary lung tumor in any given individual [36, 37]. Additionally, Graziano P et al. have reported the co-presence of lesions positive for T790M mutation with lesions that are negative in the same patients with EGFR-mutant NSCLC after development of resistance to EGFR-TKIs [38]. Although T790M mutation was previously thought to be acquired secondarily upon exposure to EGFR-TKI, recent studies have shown that subclones harboring T790M pre-exist in NSCLC patients even before EGFR-TKI treatment [39, 40]. It has been hypothesized that EGFR-TKI treatment may cause selection of cells harboring T790M mutation, even though they may represent only a small subclone of cancer cells upon initiation of treatment, consequently leading to EGFR-TKI resistance as a result of selective increase of such T790M clones [40]. T790M mutations exists as dominant clones in about 50% of patients with acquired resistance, and subclonal populations of *EGFR* mutant tumor cells with or without the T790M mutation can coexist in *EGFR* mutant NSCLC that has acquired resistance to EGFR-TKIs. A tumor sample obtained using only a single biopsy may not necessarily reflect the dominant properties of the tumor, and the primary activating *EGFR* mutation appears to represent the non-dominant tumor clone in patients with EGFR-TKI-resistant NSCLC. Indeed, primary activating *EGFR* mutations in plasma were not detected in third part of cases, even though they were detected in tumor re-biopsy samples. This mutational heterogeneity of tumors might account for the inconsistency between analysis of the primary active mutation and T790M mutation in identical paired samples, suggesting that analysis of blood samples mirrors the dominant properties of the tumor. Our results indicating that plasma samples can be used to detect T790M mutation may provide some patients with an opportunity to receive third-generation EGFR-TKIs.

The *MET* and *HER2* genes have been reported one of the mechanism of acquired resistance to EGFR-TKIs [9, 10]. Although some studies have investigated *HER2* amplification in plasma cfDNA from patients with breast cancer [41–43], no previous study has focused on *MET* and *HER2* in plasma of NSCLC patients. Therefore, in the present study, we attempted to detect *MET* and *HER2* amplifications in both tumor and plasma samples. Our analysis of tumor samples using digital PCR confirmed *MET* copy number gain in three patients. However, FISH analysis demonstrated only one patient with MET amplification, and none of the patients had a gain in *MET* copy number on the basis of plasma samples. Both *MET* gene copy number amplification and T790M mutation were detected in case 16. Both genes of these genes are considered to be exclusively involved in the mechanism of resistance to EGFR-TKIs, and these results suggest intratumonal heterogeneity such as the co-presence of clones with *MET* copy number gain and T790M mutation. The discrepancy between *MET* copy number gain and T790M mutation in the same case was considered attributable to differences in the detection sensitivity of the assays. Whereas T790M mutation is defined on the basis of detection of positivity for the mutant allele, *MET* copy number gain is defined by the degree of copy number gain relative to the reference gene. Therefore, admixing of normal cells would influence the detection of *MET* copy number gain, unlike detection of T790M mutation. Although our results did not allow us to conclude whether digital PCR is helpful for detection of *MET* and *HER2* amplification in plasma samples, we believe that a further large-scale study might clarify this issue.

In conclusion, we have demonstrated that digital PCR analysis of T790M mutation in plasma samples had high sensitivity and specificity in NSCLC patients with resistance to EGFR-TKIs. Although our study was retrospective in nature and had a relatively small sample size, our results suggest that digital PCR to detect T790M mutation in plasma samples could be an alternative and non-invasive method for patients with EGFR-positive NSCLC resistance to first- or second-generation EGFR-TKIs, especially patients from whom tumor lesions can be collected safely. In the future, plasma cfDNA may become a standard detection modality for analysis of EGFR mutations including T790M mutation.

## MATERIALS AND METHODS

### Patients

We retrospectively screened 152 consecutive EGFR mutation-positive NSCLC patients who had received EGFR-TKI treatment at Kurume University Hospital between 2007 and 2014. Among these patients, both tumor specimens and blood samples were obtained from 18 with appropriate tumor DNA after acquisition of resistance to EGFR-TKI treatment.

The present study was conducted in accordance with the provisions of the Declaration of Helsinki, and was approved by the Institutional Review Board of Kurume University Hospital.

### Sample collection and processing

We used formalin-fixed, paraffin-embedded sections or fresh frozen samples of tumors after acquisition of resistance to treatment with EGFR-TKIs. For plasma samples, 7 to 10 mL of peripheral blood was collected in heparin-coated tubes from patients after they had acquired resistance to EGFR-TKI treatment. Plasma was removed by centrifugation at 1000 rpm for 15 min within two hours of collection and stored at −80°C until DNA extraction. Plasma DNA was purified using a QIAamp Circulating Nucleic Acid Kit (Qiagen, Valencia, CA) in accordance with the manufacturer's instructions. The extracted DNA was stored at 4°C until analysis.

### Digital PCR analysis

Mutant allele frequency was measured using the QX100 Droplet Digital PCR System in accordance with the manufacturer's instructions (Bio-Rad, Hercules, CA). The primers and probes for detecting EGFR E746-A750 deletion, L858R, and T790M were purchased from Bio-Rad. The PCR reaction was performed using the following cycling conditions: 95°C for 10 min, 40 cycles of 94°C for 30 s and 55°C for 60 s, followed by enzyme deactivation at 98°C for 10 min. For MET copy number assay, the primer sequences were: MET forward, 5′-TTAGTTCGCTACGATGCAAGAG-3; MET reverse, 5′-GGCTTACACTTCGGGCACT-3′; MET probe, 5′-/56- FAM/CACACTCCT/ZEN/CATTTGGATAGGCTTG/3IB κFQ/-3′; RPP30 forward, 5′-GATTTGGACCTGCGAG CG-3′; RPP30 reverse, 5′-GCGGCTGTCTCCACAA GT-3′; RPP30 probe, 5′-/5HEX/CTGACCTGAAGGCT CT/3IABkFQ/-3′. The PCR reaction was performed using the following cycling conditions: 95°C for 10 min, 40 cycles of 94°C for 30 s and 60°C for 90 s, followed by enzyme deactivation at 98°C for 10 min. For HER2 copy number assay, the primer sequences were: HER2 forward, 5′-ACAACCAAGTGAGGCAGGTC-3′; HER2 reverse, 5′-GTATTGTTCAGCGGGTCTCC-3′; HER2 probe, 5′-/56-FAM/AGGCACCC A/ZEN/GCTCTTTGAGGA CAAC/3IABkFQ/-3′; EFTUD2 forward, 5′-GGTCTTGCC AGACACCAAAG-3′; EFTUD2 reverse, 5′-TGAGAGGA CACACGCAAAAC-3′; EFTUD2 probe, 5′- /5HEX/TC CAGGTAG/ZEN/GACATCCTTTGGCTTT/3IABkFQ/-3′ [30]. The PCR reaction was performed using the following cycling conditions: 95°C for 10 min, 40 cycles of 94°C for 30 s and 58°C for 90 s, followed by enzyme deactivation at 98°C for 10 min. After thermal cycling, the plates were transferred to a Droplet reader. The digital PCR data were analyzed using the QuantaSoft analytical software package (Bio-Rad). We have used control plasmids encoding wild-type *EGFR*, E746-A750 deletion, L858R, and a T790M mutant as a control. The assay has been validated to detect mutant variants at a level of 0.032%.

The cutoff values for DNA derived from the FFPE specimens were determined using data for mutation-negative FFPE samples. The average and standard deviation (SD) for EGFR E746-A750 deletion, L858R, and T790M mutant copy number in 21 FFPE samples were calculated. The average copy number for negative samples plus 3SD was used as the cut-off value for each mutation site; the higher value of either, the average plus 3SD or three copies was used as the cut-off value. The cut-off values were set at 6.0 copies for EGFR E746-A750, 4.0 copies for EGFR L858R, and 22.0 copies for EGFR T790M. The cut-off values for DNA derived from plasma or frozen samples were determined using data for 10 normal DNA samples. The cut-off values were set at 3.0 copies for EGFR E746-A750, L858R, and T790M. MET and HER2 gene copy numbers were normalized against RPP30 and EFTUD2, respectively. Normal genomic DNA (Promega, Madison, WI) was used as the normal control (two copies). The cut-off values were set at 5.0 copies for MET and HER2 copy number gain.

### Fluorescence in situ hybridization for MET

Fluorescence in situ hybridization (FISH) was performed to determine the MET copy number in FFPE tumor specimens using a c-Met/CEN7p Dual Color FISH Probe (GSP Laboratory, Kawasaki, Japan), where CEN7p is the centromeric region of chromosome 7p. After screening of all sections, images of tumor cells were captured and recorded, and the signals for at least 50 random nuclei were counted for an area in which individual cells were recognized in each of at least 10 representative images. Nuclei with a disrupted boundary were excluded from the analysis. Gene amplification was strictly defined on the basis of a mean MET/CEN7p copy number ratio of >2.2, as described previously [44].

### Analysis for sensitivity and specificity

Sensitivity was defined as the probability of detecting plasma positive positivity findings in plasma among patients with for whom the presence of the mutation had been confirmed in tumor samples, and whereas specificity was defined as the probability to of detecting plasma negative negativity in plasma among patients without who did not harbor the mutation in tumor samples.
